# Cross-Cultural Adaptation and Validation of the Serbian Version of the ICS SF Male Questionnaire

**DOI:** 10.1155/2015/673196

**Published:** 2015-02-15

**Authors:** Uros Babic, Milena Santric-Milicevic, Vesna Bjegovic-Mikanovic, Aleksandar Argirovic, Mihailo Stjepanovic, Dejan Lazovic, Djordje Nale, Milan Perovic, Stefan Dugalic, Vinka Vukotic

**Affiliations:** ^1^Clinical Center of Serbia, Pasterova 2, 11000 Belgrade, Serbia; ^2^Institute of Social Medicine, Dr Subotica 15, 11000 Belgrade, Serbia; ^3^Faculty of Medicine, University of Belgrade, Dr Subotica 8, 11000 Belgrade, Serbia; ^4^Clinical Center “Zemun”, Vukova 9, 11070 Belgrade, Serbia; ^5^Clinic for Gynecology and Obstetrics, Narodni Front, Kraljice Natalije 62, 11000 Belgrade, Serbia; ^6^Clinical Center “Dr Dragisa Misovic-Dedinje”, Heroja Milana Tepica 1, 11000 Belgrade, Serbia

## Abstract

*Introduction*. The objective of this study was to cross-culturally adapt and validate ICS male SF questionnaire to Serbian language. *Materials and Methods*. This study included 91 male patients with lower urinary tract symptoms and 24 men with similar age and with confirmed absence of LUTS. ICS male SF questionnaire was translated from English to Serbian language and then back-translated to English. *Results*. Internal consistency was high in both dimensions, voiding (Cronbach's alpha = 0.916) and incontinence (Cronbach's alpha = 0.763). Comparison of the average scores between patients and controls revealed significant differences in both dimensions: voiding (med = 8 versus med = 0; *P* < 0.001) and incontinence (med = 3 versus med = 0; *P* < 0.001). Interclass correlation revealed high testretest validity in both dimensions, voiding ICC = 0.992 (*P* < 0.001) and incontinece ICC = 0.989 (*P* < 0.001). Correlation analysis revealed high agreement between ICS male SF voiding dimension and IPSS questionnaire (*ρ* = 0.943; *P* < 0.001). *Conclusion*. The Serbian version of male ICS SF questionnaire showed acceptable reliability and validity. The ICS male SF questionnaire could be used in routine practice as an easy and comprehensive tool for assessment of LUTS.

## 1. Introduction

Due to the aging of the world population, the lower urinary tract symptoms (LUTS) have become a prevalent problem in today's urological practice [1, 2].

Though not life-threatening, these symptoms significantly affect patient's life quality, mental and physical condition, labor productivity, and sexual function [3–5]. LUTS are staged into storage, voiding, and postmicturition [6]. Storage symptoms comprise nocturia, urgency, increased micturition frequency, and urinary incontinence which is an involuntary urine loss and presents a great burden on life quality. Voiding symptoms include weak stream, intermittent flow, hesitancy, and straining. Postmicturition symptoms are a sensation of incomplete emptying and a postmicturition dribble. Benign prostatic hyperplasia (BPH), urethral stenosis, urethral hypermobility, stones and cancer in bladder, and detrusor overactivity are the most common causes of LUTS in male population [3]. There are numerous questionnaires for the assessment of LUTS caused by BPH and their impact on quality of life: IPSS [7], DAN-PSS-1 [8], BPH impact index [9], Bolognese questionnaire [10], AUA symptom score [11], and ICS SF male questionnaire [12], but besides IPSS, for none of the questionnaires was the validation done in Serbian language. ICS male SF questionnaire was developed and validated as an interviewer-administered and self-administered instrument assessing storage, voiding, and postmicturition symptoms and their impact on the quality of life (QOL). Validation of this questionnaire, which is more complete than the IPSS score, represents an important instrument for the assessment of the lower urinary tract symptoms and their impact on the patient as well as the need for conservative or operative treatment.

## 2. Materials and Methods

Study was approved by the Ethics Committee of the Clinical Center “Dr Dragisa Misovic-Dedinje,” Belgrade, Serbia. First, copyright form of ICS male SF questionnaire was authorized from the author of original questionnaire. Then, the forward translation was done. ICS SF male questionnaire was translated from English into Serbian by two translators. Both are native Serbian speakers and have done translation independently from each other. After the questionnaire was translated, both versions of the translation were reviewed by both translators and research team and the final version of questionnaire in Serbian language was done. This version was again translated back to English by a professional bilingual translator who is a native English speaker, and the new English version of Serbian questionnaire was done (back translation). Original version was compared with the back-translated version and after the differences were analyzed, the research team concluded that no significant difference between these two questionnaires existed.

After translation, pilot study was done in order to assess any misunderstanding and suggestions from patients. Some questions were restructured according to patient's suggestions. Research team again revised the questionnaire and improved the understandability and the final version of ICS male SF questionnaire was then used for validation.

The study was conducted from May 2013 to March 2014 in the Clinical Center “Dr Dragisa Misovic-Dedinje” in Belgrade, Serbia. In this study, consecutive male patients with lower urinary tract symptoms were included. Inclusion criteria were male gender, age above 18, and presence of lower urinary tract symptoms. Exclusion criteria were cognitive disorder or mental incapacity that compromises completion of the questionnaire. LUTS were defined as presence of voiding, incontinence, and postmicturition symptoms. The control group included 24 men with similar age and with confirmed absence of LUTS. All patients, including healthy controls, were informed about study objectives and the written informed consent was obtained from all participants. The number of controls (*n* = 24) was sufficient to detect significant difference between patients and controls with study power being more than 80% (1 − *β* = 0.80).

Serbian version of ICS male SF questionnaire was evaluated by using statistical procedures to assess internal consistency, construct validity, test-retest reproducibility, and discriminant validity. Internal consistency was analyzed using Cronbach's alpha and values of 0.7 or more were considered as high internal consistency of items [13].

Intraclass correlation coefficient (ICC) was used to assess test-retest reliability of scores [14]. Since those patients were in hospital, the interval of 4 days appeared long enough to avoid any confusion with previous testing. No surgical treatment has been done prior to testing. Control group and patients were compared (ICS male SF questionnaire) to assess discriminant validity.

Confirmatory factor analysis (CFA) was performed to test factor structure based on the original construct. Questionnaire has 11 questions divided into two separate scores (5 for voiding and 6 for incontinence). Voiding and incontinence dimensions (factors) should be highly correlated (expected since voiding problems correlate with incontinence and vice versa). Since ICS male SF questionnaire was developed with a priory hypothesized relationship among items, CFA is adequate utilization tool for assessing construct validity of this questionnaire. Goodness of fit is expressed by Comparative Fit Index (CFI), root mean square error of approximation (RMSEA), and chi-square test. CFI is calculated by subtracting df from chi-square. Values higher than 1 are truncated to 1 and values less than 0 are raised to 0. Cut-off value of ≥0.95 is suggested as value for a good fit [15, 16]. RMSEA is based on chi-square, df, and *N* [17]. Dividing by df, RMSEA penalizes free parameters and also rewards a large sample size, since *N* is in the denominator. A value of 0 indicates perfect fit but suggested value is ≤0.06. The difference between matrices (expected and observed covariance matrices) is expressed as overall goodness of fit chi-square, with degrees of freedom (df) equaling the number of covariances in the matrix minus the number of free parameters. Chi-square value, in relation to df, should be small enough to be nonsignificant to indicate good fit. Value close to zero indicates small differences between matrices. But chi-square test is not robust enough because with large samples even small differences may be significant, suggesting poor fit. Some researchers assess goodness of fit by chi-square to df ratio (CMIN/DF). Suggested ratio for good fit is less than 3, somewhat 5. The modification index reflects an approximation of how much the overall model chi-square will decrease if the fixed or constrained parameter is freely estimated [18]. Large modification indices are obtained in regard to correlated errors of items (items 9 and 10). Consequently, covariance was drawn between error 9 and error 10.

To archive minimum sample size required for the analysis, we used 10 : 1 recommended ratio (10 cases per variable) and minimum 100 cases. Maximum likelihood estimation is used as standard estimation method.

Results are presented as number of patients and percent (*n*, %), mean ± standard deviation, or median (minimum-maximum). Differences between the groups were analyzed using *t*-test and Mann-Whitney *U* test. Correlations between variables were assessed using Spearman correlation analysis. Confirmatory factor analysis was used to examine hypothesized theoretical model using AMOS 18 (IBM Corp.). All other data were analyzed using SPSS 20.0 (IBM Corp.). All *P* values less than 0.05 were considered significant.

## 3. Results

Basic characteristics, age, BMI, and time to complete the questionnaire, are presented in Table 1.

According to basic characteristics of patients and controls, average age and BMI were similar in both groups, with no statistically significant differences. Also time to complete questionnaire was almost identical in both groups.

Cronbach's alpha was high in both dimensions, voiding and incontinence, but higher in voiding dimension. Also, “alpha if item deleted” and “correlation between score and items” were higher in voiding dimension than in incontinence (Table 2).

Comparison of the average scores (voiding and incontinence) between patients and controls revealed significant differences. According to our results, patients had significantly higher scores than controls (Table 3).

Test-retest validity was done using ICC and the results are presented in Table 4. As shown in Table 4, medians of voiding and incontinence were almost identical on baseline and four days after. High ICC with high significance confirmed test-retest validity of questionnaire.

In order to assess agreement of examined questionnaire (ICS male SF) and gold standard (IPSS score), Spearman correlation was done and high coefficient (*ρ* = 0.943; *P* < 0.001) confirmed high degree of agreement between these two scores. Unfortunately, no valid questionnaire for male incontinence exists in Serbian medical practice, so there is no possibility to explore agreement between incontinence score of ICS male SF questionnaire and gold standard.

But IPSS and ICS male SF questionnaire have almost identical three questions: urinating frequency during day, nocturia, and quality of life due to urinary symptoms. Correlation analysis revealed high agreement between these two questionnaires. Correlation of urination frequency during day was *ρ* = 1.000; *P* < 0.001; urination frequency during night was *ρ* = 0.941; *P* < 0.001; and quality of life due to urinary symptoms was *ρ* = 1.000; *P* < 0.001, which points out high level of agreement.

**Figure 1:**
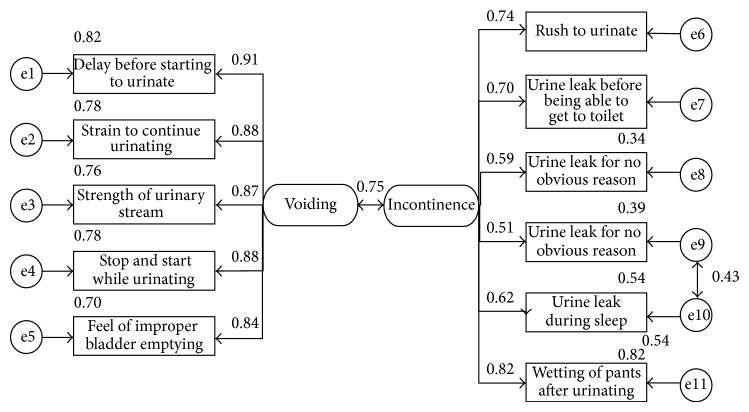
Confirmatory factor analysis of voiding and incontinence symptoms.

Confirmatory factor analysis was conducted on a model representing the items and the corresponding factors (Figure 1). Factor loadings varied from 0.84 to 0.91 in voiding dimension, and much higher variation was observed in incontinence dimension, from 0.59 to 0.72. High correlation was observed between these two factor scores.

Even though chi-square test revealed significance, chi-square to df is less than 3 (chi-square = 85.2; df = 42; *P* < 0.001; CMIN/DF = 2.029), CFI is satisfying (CFI = 0.947), and RMSEA is higher than 0.05 but lower than 0.1 (RMSEA = 0.085) which could be classified as moderate.

## 4. Discussion

According to our findings, beside IPSS this is the first instrument for assessment of voiding symptoms with incontinence symptoms in Serbian male population. IPSS is widely used in Serbian medical practice, but its lack of incontinence evaluation makes us look for a more comprehensive questionnaire for assessment of all dimensions of LUTS and, subsequently, quality of life.

High similarity between languages in Balkan (six countries of former Yugoslavia) region also emphasizes importance of Serbian version of ICS male SF questionnaire.

The translation process included two forward translations, reconciliation, and one backward translation with cognitive debriefing. It was all part of linguistic validation which does not include simple translation from English to Serbian language. Other studies which validated similar questionnaires were similar to our study [19].

Cronbach's alpha was used to analyze internal consistency of dimensions of Serbian version of ICS male SF questionnaire. As shown in Table 2, much higher internal consistency was observed in voiding dimension, compared to incontinence. In the original article, internal consistency was similar to our results, voiding symptoms alpha = 0.76, and incontinence symptoms alpha = 0.78. In our study, internal consistency of voiding symptoms is higher than that in original study, but incontinence is very similar [20]. Test-retest reliability analyzed with ICC showed correlation above 0.9, which was regarded as acceptable. In original study, test-retest reliability analysis revealed similar results of our study to those of the original one with 0.04 mean change in voiding score and 0.3 in incontinence score.

Significant difference was observed between patients and controls in voiding and incontinence scores. This emphasizes discriminant validity of ICS male SF questionnaire as a good tool to discriminate patients from healthy subjects.

Confirmatory factor analysis showed that data has shown good but not excellent fit to hypothesized model of the Serbian version of ICS male SF questionnaire but the reason could be small sample size for this analysis. But if we neglect lack of model fit, original theoretical model could be applied for the Serbian version with appropriate construct validity. But our model revealed more compact structure of voiding than incontinence. From this analysis, it was observed that the items share common latent construct and high associations between latent variables (based on the factor loadings and correlation). Also, correlation between the factors is very high, showing that subscales measures similar pathology in different symptomatology.

## 5. Conclusion

In order to provide questionnaire identical to the original English version, ICS male SF questionnaire was successfully translated into Serbian language. The Serbian version of male ICS SF questionnaire showed acceptable reliability and validity. The ICS male SF questionnaire could be used in routine practice as an easy and comprehensive tool for assessment of LUTS including voiding and incontinence symptoms together.

## Limitations

The limitation of this study is the fact that there is no validated questionnaire for incontinence in Serbian language to assess convergent validity of incontinence dimension of ICS male SF questionnaire. Also, diagnosis of LUTS was based on anamnestic data only without urodynamics.

## Figures and Tables

**Table 1:** Basic characteristics of patients and controls.

	Patients (*n* = 91)	Controls (*n* = 24)	*P* value
Age	67.3 ± 14.4	64.9 ± 17.1	0.486
BMI	27.4 ± 5.8	29.1 ± 6.5	0.215
Time to complete (min)	10.3 ± 1.2	9.9 ± 1.4	0.163

**Table 2:** Validity of voiding and incontinence scores.

Score	Cronbach's alpha	Alpha if item deleted	Correlation between score and items
Voiding	0.916	0.888–0.910	0.777–0.898
Incontinence	0.763	0.696–0.757	0.500–0.797

**Table 3:** Known-group validity.

Score^*^	Patients (*n* = 91)	Controls (*n* = 24)	*P* value^a^
Voiding	8 (0–20)	0 (0–2)	<0.001
Incontinence	3 (0–14)	0 (0-1)	<0.001

^a^Mann-Whitney *U* test; ^*^results are presented as median (min-max).

**Table 4:** Test-retest validity.

ICS score	*n* = 23		
	Baseline	Four days after	ICC^a^ (*P* value)
Voiding	10 (0–19)	11 (0–19)	0.992 (<0.001)
Incontinence	3 (0–13)	3 (0–12)	0.989 (<0.001)

^a^Intraclass correlation coefficient (ICC).

## Appendix

### A. Serbian Version of the ICS SF Male Questionnaire

Упитник се односи на Ваше евентуалне симптоме (ПРОБЛЕМЕ) са мокрењем. Молимо вас да одговорите на свако питање, имајући у виду симптоме које сте имали током претходног месеца.

Понуђeни су Вам одговори на нeка питања о  томe колико чeсто иматe симптомe:
Поврeмeно = мањe од јeднe трeћинe врeмeна (дана)
Понeкад = измeђу јeднe и двe трeћинe врeмeна (дана)
Чeсто = вишe од двe трeћинe врeмeна (дана).

Молим вас штиклирајтe јeдно пољe за свако питањe ✓

B1 Да ли на почeтку мокрeња чeкатe да млаз крeнe?
  **Table:**
  никад	MZ 0
  поврeмeно	MZ 1
  понeкад	MZ 2
  чeсто	MZ 3
  увeк	MZ 4
B2 Да ли сe напрeжeтe током мокрeња?
  **Table:**
  никад	MZ 0
  поврeмeно	MZ 1
  понeкад	MZ 2
  чeсто	MZ 3
  увeк	MZ 4
B3 Да ли јe ваш млаз током мокрeња…
  **Table:**
  нормалан	MZ 0
  поврeмeнослаб	MZ 1
  понeкадслаб	MZ 2
  чeстослаб	MZ 3
  увeкслаб	MZ 4
B4 Да ли вам сe млаз прeкида током мокрeња?
  **Table:**
  никад	MZ 0
  поврeмeно	MZ 1
  понeкад	MZ 2
  чeсто	MZ 3
  увeк	MZ 4
B5 Колико чeсто иматe утисак да нистe испразнили бeшику до краја?
  **Table:**
  никад	MZ 0
  поврeмeно	MZ 1
  понeкад	MZ 2
  чeсто	MZ 3
  увeк	MZ 4
И1 Да ли моратe да журитe у тоалeт како бистe мокрили?
  **Table:**
  никад	MZ 0
  поврeмeно	MZ 1
  понeкад	MZ 2
  чeсто	MZ 3
  увeк	MZ 4
И2 Да ли вам мокраћа капљe/цури прe нeго што стигнeтe до тоалeта?
  **Table:**
  никад	MZ 0
  поврeмeно	MZ 1
  понeкад	MZ 2
  чeсто	MZ 3
  увeк	MZ 4
И3 Да ли вам мокраћа цури када кашљeтe или кијатe?
  **Table:**
  никад	MZ 0
  поврeмeно	MZ 1
  понeкад	MZ 2
  чeсто	MZ 3
  увeк	MZ 4
И4 Да ли вам мокраћа цури бeз посeбног разлога и бeз осeћаја да вам сe идe у тоалeт?
  **Table:**
  никад	MZ 0
  поврeмeно	MZ 1
  понeкад	MZ 2
  чeсто	MZ 3
  увeк	MZ 4
И5 Да ли вам мокраћа цури у току спавања?
  **Table:**
  никад	MZ 0
  поврeмeно	MZ 1
  понeкад	MZ 2
  чeсто	MZ 3
  увeк	MZ 4
И6 Колико чeсто вам сe дeшавало да овлажитe вeш нeколико минута након мокрeња и облачeња?
  **Table:**
  никад	MZ 0
  поврeмeно	MZ 1
  понeкад	MZ 2
  чeсто	MZ 3
  увeк	MZ 4
Учeсталост Колико чeсто мокритe у току дана?
  **Table:**
  сваког сата	MZ 0
  свака 2 сата	MZ 1
  свака 3 сата	MZ 2
  свака 4 сата или вишe	MZ 3
Ноћнаучeсталост У току ноћи, колико пута у просeку моратe да устајeтe како бистe мокрили?
  **Table:**
  нијeдном	MZ 0
  јeдном	MZ 1
  два пута	MZ 2
  три пута	MZ 3
  чeтири или вишe пута	MZ 4
QоЛ Свe у свeму, колико начин мокрeња утичe на ваш живот?
  **Table:**
  нe уопштe	MZ 0
  помало	MZ 1
  прилично	MZ 2
  много	MZ 3
Молим вас упишитe данашњи датум
MZMZ MZMZ MZMZ
